# Function, clinical application, and strategies of Pre-mRNA splicing in cancer

**DOI:** 10.1038/s41418-018-0231-3

**Published:** 2018-11-21

**Authors:** Cuixia Di, Syafrizayanti, Qianjing Zhang, Yuhong Chen, Yupei Wang, Xuetian Zhang, Yang Liu, Chao Sun, Hong Zhang, Jörg D. Hoheisel

**Affiliations:** 1 Department of Radiation Medicine, Institute of Modern Physics, Chinese Academy of Sciences, 730000 Lanzhou, China; 2 Key Laboratory of Heavy Ion Radiation Biology and Medicine of Chinese Academy of Sciences, 730000 Lanzhou, China; 3 College of Life Sciences, University of Chinese Academy of Sciences, Beijing, China; 4 Division of Functional Genome Analysis, German Cancer Research Center (DKFZ), Im Neuenheimer Feld 580, 69120 Heidelberg, Germany; 5 Department of Chemistry, Faculty of Mathematics and Natural Sciences, Andalas University, Kampus Limau Manis, Padang, Indonesia

**Keywords:** Genetics, Cancer, Cancer genetics

## Abstract

Pre-mRNA splicing is a fundamental process that plays a considerable role in generating protein diversity. Pre-mRNA splicing is also the key to the pathology of numerous diseases, especially cancers. In this review, we discuss how aberrant splicing isoforms precisely regulate three basic functional aspects in cancer: proliferation, metastasis and apoptosis. Importantly, clinical function of aberrant splicing isoforms is also discussed, in particular concerning drug resistance and radiosensitivity. Furthermore, this review discusses emerging strategies how to modulate pathologic aberrant splicing isoforms, which are attractive, novel therapeutic agents in cancer. Last we outline current and future directions of isoforms diagnostic methodologies reported so far in cancer. Thus, it is highlighting significance of aberrant splicing isoforms as markers for cancer and as targets for cancer therapy.

## Facts

- Differential splicing is an important factor of expanding and diversifying the molecular function portfolio of human cells.
- Cancer-associated splicing events have been found to be involved in human cancer initiation and tumour development.
- Splice differences can be exploited as markers for more accurate diagnosis as well as improved prognosis and monitoring of treatment response in patients with cancer.
- Technology has facilitated the investigation of splicing changes; rather than getting isolated results from distinct studies, a more global picture of the role of alternative RNA splicing in human cancer is beginning to emerge.

## Open Questions

- Do personal splice variations or particular combinations thereof exist in individual cancer patients?
- What are the strategies modulate target gene splicing and thus inhibit tumour growth of human cancer?
- Can protein isoforms that results from alternative RNA splicing in individual patients with cancer provide treatment options as part of a precision medicine approach?
- Could splice variations be better reversed by applying new genome editing processes, such as CRISPR-Cas, or would proteomic procedures, such as provision of specific antibodies against the tumour-specific protein isoform do a better and/or technically superior job in clinical application?

## Introduction

Most human genes harbour introns that are removed during pre-mRNA splicing [1]. Next-generation sequencing results suggest that more than 90% of human genes that encode proteins undergo pre-mRNA splicing [2]. Pre-mRNA splicing is a common post-transcriptional process used by eukaryotic organisms to generate multiple transcript isoforms from a single gene. This process expands substantially the variety of encoded proteins, thus providing another means of functional regulation [3]. Structural differences that result from splice differences are likely to translate to functional variation. Regulation of splicing could therefore affect cellular fate and function in cancer [4].

**Fig. 1:**
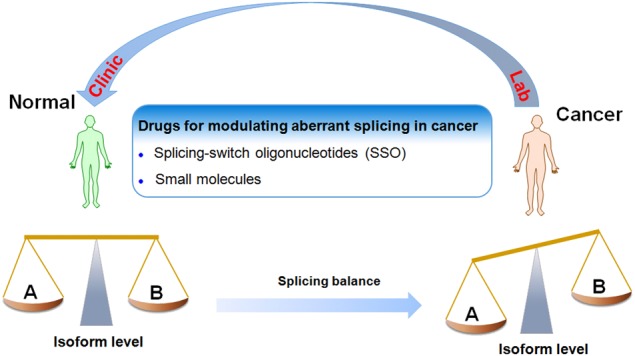
The role of alternative RNA splicing in cancer. The balance between noncancerous isoform (**a**) and cancerous isoform (**b**) is an important contributor to cancer genesis. To date, there are particularly two approaches of interfering with the RNA splicing process: the use of SSO and application of appropriately active small molecules

Pre-mRNA splicing has been discovered to be critical for genesis and development of different types of cancer [5, 6]. An increasing body of data points to a role of pre-mRNA splicing in controlling the switch between cell life and death in cancer (Fig. 1). The balance between noncancerous isoform and cancerous isoform is an important contributor to cancer genesis. To date, there are particularly two approaches of interfering with the RNA splicing process. Hence the aim of this review is to discuss how alternative splicing precisely regulates proliferation, metastasis, apoptosis, drug resistance and radiosensitivity in cancer. Additionally, we discuss emerging novel strategies how to modulate pathologic splicing isoforms in cancer. For any pharmacological regulation, this would create the opportunity to produce a drug that may affect the tumour in a really targeted manner. Furthermore, we outline current and future directions of methodologies reported so far for measuring aberrant splicing isoforms. Technological developments could create a knowledge basis rather quickly, which is sufficiently large and solid enough to identify aberrant splicing isoforms as markers for cancer. Thus this review elaborated from different angles will be helpful to better understand the significance of alternative splicing as markers for cancer and targets for future therapeutic strategies.

## Types of AS and its regulation

**Fig. 2:**
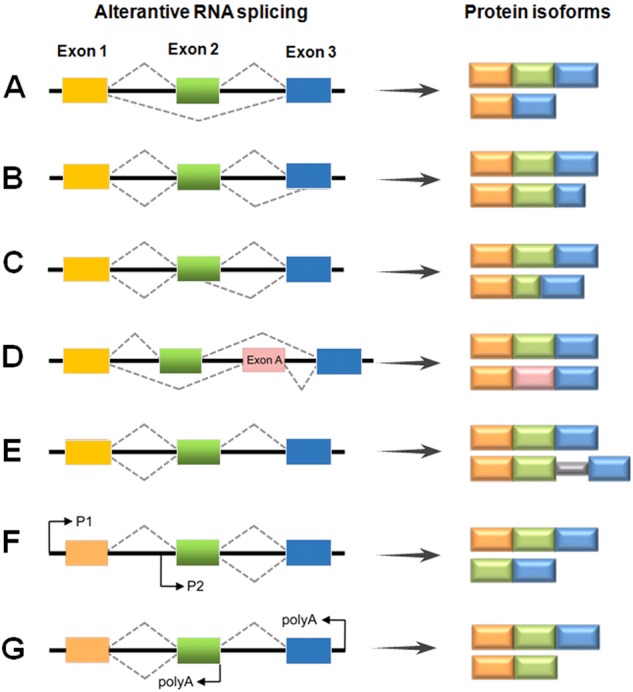
Schematic representation of different types of RNA splicing. The dotted grey lines indicate the alternative splicing processes: **a** exon skipping; **b**, **c** alternative 3′- and 5′-SS; **d** mutually exclusive exons; **e** intron retention; **f** usage of alternative promoters; **g** alternative polyadenylation. P promoter, polyA site of polyadenylation

While many exons are constitutively spliced together, alternative splicing (AS) is a process during which specific exons are selectively included or excluded [7]. AS is of great physiological relevance as it enables the production of multiple protein isoforms from a single pre-mRNA molecule by the combinatorial use of splice sites. This gives rise to a variety of proteins that might show similar, different or even opposing functions. In addition to protein diversity, AS can also lead to reduced translation of mRNAs through introduction of a premature stop codon leading to sequestration and degradation of transcripts in the nucleus; these exons are often referred to as poison exons [8, 9]. There are different types of alternative splicing events, which generate mRNAs encoding proteins (Fig. 2). The most common type of AS, accounting for nearly 40% of events in higher eukaryotes is exon skipping [10], in which a cassette exon is spliced out of the transcript together with its flanking introns (Fig. 2a). One or several exons are not present in the final mRNA. Second and third, there are alternatives in the selection of either 3′- or 5′-splice sites (Fig. 2b, c). The fourth type of splice variation is mutually exclusive exons (Fig. 2d), which represents a rare subtype [11]. The fifth type of splice variation is intron retention (Fig. 2e), in which an intron remains in the mature mRNA transcript rather than being removed during maturation. RNA sequencing and other data demonstrate that intron retention is a common mechanism of tumour-suppressor inactivation, is widespread across cancer entities and contributes to their transcriptional diversity [12]. There are also other types of AS, include use of alternative promoters, and unusual polyadenylation (Fig. 2f, g). Recently, it was shown that exon transcripts in pre-mRNAs might also be non-linearly reverse-spliced into a circular RNA [13].

The chemistry of the splicing reaction is mediated by the “spliceosome”, an RNA-based machine containing five small nuclear ribonucleoproteins snRNAs and numerous associated proteins [14]. Splicing regulation involves both cis-acting splicing-regulatory elements (SREs) and trans-acting splicing factors [15]. SREs act as either enhancers or silencers of splicing and can exist in either exons or introns. Historically these elements are named as exonic splicing enhancers or silencers, and intronic splicing silencers or enhancers. By recruiting trans-acting splicing factors, cis-acting splicing-regulatory elements either activate or inhibit the usage of adjacent splice sites, thus to specifically control alternative splicing [16]. Changes in the activity and composition of general or specific splicing factors modify the selection of splice sites [17]. Recent reports showed that DNA methylation in either cis-acting splicing-regulatory elements or trans-acting splicing factors is associated with various cancers, such as prostate [18], breast, lung cancer [19] and et al. Whilst extensive studies reported that single-nucleotide polymorphisms in these sites could modulate the risks of cancer development and outcome [20].

## Function of aberrant splicing isoforms in cancer

For a number of relevant genes, RNA splicing determines isoforms with distinct and even opposing functions (Table 1). Some RNA splicing isoforms are listed here to show how precisely they regulate proliferation, metastasis, and apoptosis in cancer. Various splicing events are not separated, but co-ordinated in cancer cell.

**Table 1:** Aberrant splicing isoforms in cancer and its clinical application

	Isoform A	Function	Cancer type	Splicing type	Refs
	Isoform B				
Proliferation	cyclin D1	Tumour suppressor gene	Prostate tumours	Intron retention	[21]
	cyclin D1b	Associate with progression			
	Syk	Tumour suppressor	Breast tumours	Exon skipping	[26]
	Syk(S)	Mammary tumour progression			
	RASSF5	Tumourigenesis	Oesophageal cancer	Promoter usage	[25]
	RASSF5A	Tumour suppressor			
	WT1	Proliferation	Breast cancer	Exon skipping	[27, 100]
	WT1iso	Development & progression			
Metastasis	CD44	Drive metastasis	Breast cancer	Addition of amino acids	[30]
	CD44V6	Pro-metastatic molecule			
	CrkII	Migration	Glioblastoma	Exon skipping	[101]
	CrkI	Promote cell migration			
	KLF6	Tumour suppressor gene	Lung cancer	5′- SS	[34]
	KLF6-SV1	Driver of tumour metastasis			
	VEGFA	Metastasis	Sarcoma	Exon 8	[35]
	VEGFA iso	Advantage for metastasis			
Apoptosis	BCL-Xs	Pro-apoptosis	Many cancer types	5′-SS	[75]
	BCL-XL	Anti-apoptosis			
	nCLu	Pro-apoptotic	Many cancer types	Frame shift	[41]
	sCLu	Pro-survival			
	ELF2A	Pro-apoptosis	Cancer cell lines	5′-SS	[42]
	ELF2B	Anti-apoptosis			
	RIP3	Pro-apoptosis	Colon & lung cancer	Intron retention	[43]
	RIP3γ	Anti-apoptosis			
Drug resistance	AR	Chemosensitivity	Prostate cancer	Exon skipping	[70]
	AR-v7	Chemoresistance			
	BRCA1	Repair of DNA break	Breast cancer	Exon skipping	[49]
	BRCA1Δ11	Chemoresistance			
	c-FLIP(L)	Anti-apoptosis	Pancreatic cancer	Truncated C-terminus	[50]
	c-FLIP(S)	Drug resistance			
	Survivin	Anti-apoptosis	Neoplastc cells	Intron retention	[48]
	Survivin 3B	Promote chemoresistance			
Radiotherapy	sCLu	Genomic instability	Breast cancer cells	5′-SS	[53]
	nCLu	Pro-death factor			
	Mcl-1L	Anti-apoptotic	OSCC	Exon skipping	[54]
	Mcl-1S	Pro-apoptotic			
	NPM1	Radioresistance	Cervical cancer	Intron retention	[55]
	NPM2	Radiosensitivity			
	Tap73	Pro-apoptosis	Cervical cancer	Exon skipping	[60]
	ΔNp73	Anti-apoptosis			

## Proliferation

Numerous cases of splicing isoforms that drive or promote cancer progression have been identified over the years. Some isoforms are selected to show how they exactly regulate proliferation in cancer (Table 1). Cyclin D1b, an isoform of cyclin D1, is originated from intron-4 retention. Clinical evidence demonstrated that cyclin D1b is induced in lymphoma, oesophageal, breast, lung and prostate cancer [21]. Furthermore, cyclin D1b expression is associated with tumour progression and therapeutic failure in breast [22] and prostate cancer [23], and is an independent predictor of poor prognosis and survival in small-cell lung cancer [24]. Thus these findings suggest that cyclin D1b represents an oncogenic isoform of cyclin D1. Also, ras-association domain family 5 isoform A (RASSF5A) originated from different promoter usage and was considered to be a candidate tumour suppressor that was epigenetically inactivated in oesophageal squamous cell carcinoma [25]. Interestingly, spleen tyrosine kinase (Syk) is a candidate tumour suppressor that is highly expressed in mammary epithelial cells. While the aberrant expression of Syk(S) occurs frequently in primary breast tumours but never in matched normal mammary tissues, suggesting a contribution of Syk(S) to mammary tumour progression [26]. Recently, another study identified an isoform of wilms’ tumour 1 (WT1)-produced by exon skipping, leading to cell proliferation, and might be involved in the development and progression of breast cancer [27]. Thereby, differential splicing is an important factor of expanding and diversifying the molecular function portfolio of proliferation in human cancer cells.

## Metastasis

Some RNA splicing isoforms are listed here to show how precisely they regulate metastasis (Table 1). One of the interesting examples is CD44. Its pre-mRNA harbours 10 adjacent exons that are included in a combinatorial manner to give rise to multiple isoforms. Switch from CD44v to CD44 standard (CD44s), leading to suppression of lung colonisation of metastatic cancer [28]. Among CD44v isoforms, CD44v6 seems has been extensively studied in different types of cancer including colon, ovarian, and head and neck [29]. CD44v6 is more specifically expressed in cancer tissues, but CD44 has an ubiquitously expression pattern [30]. CD44v6 was also found to be a marker of constitutive and reprogrammed cancer stem cells driving colon cancer metastasis [31]. Therefore, CD44v6 has attracted more interest than CD44 in terms of tumour markers, diagnosis, and treatment. In line with a potential therapeutic application, antibodies to CD44v6 have shown promise in human head and neck cancer in a clinical trial [32]. Additionally, another isoforms also exhibited similar properties like CD44. The human *Crk* gene, for instance, was translated into CrkI and CrkII. CrkII was detected in both normal brain and glioblastoma tissues, whereas CrkI levels were quite low in normal brain but up-regulated in glioblastoma tissues [33]. Additionally, *KLF6* is tumour suppressor gene, but its isoform has different role. Hatami et al. reported that the Klf6-sv1 isoform was a key driver of metastasis and could act as a potential therapeutic target for invasive breast cancer [34]. Vascular endothelial growth factor A (VEGFA) was a potent regulator that contributed to tumour growth and metastasis, while the isoform expression could be used to predict treatment outcome with bevacizumabin in sarcoma [35]. A frequent observation is that high expression of the α6 integrin subunit is a biomarker for breast and other cancer stem cells, but the α6Bβ1 integrin isoform drives cancer stem cells function in triple-negative breast cancer and promotes tumour initiation [36]. Chang et al. [37] reported that a laminin 511 matrix functions as the ligand for the α6Bβ1 integrin to sustain breast cancer stem cells. Thus, these suggest that the aberrant splicing isoforms may be therapeutic targets.

## Apoptosis

An increasing body of data points to a role of RNA splicing in controlling the switch between cell life and death in cancer [38]. One of the earliest oncogenic AS events described is the apoptosis gene Bcl-x [39], which generates two splicing isoforms, a shortform (Bcl-xS) with proapoptotic properties and a long form (Bcl-xL) with an antiapoptotic effect. A wide range of tumours, including breast, colon, and lung, exhibit high levels of Bcl-xL, resulting in reduced apoptosis potential and providing the tumour with elevated cell survival [29, 40]. Another isoforms also exhibited similar properties like Bcl-x. Clusterin (CLU) was implicated in various cell functions involved in the tumorigenesis of various malignancies, but the isoform of the CLU protein was shown to act pro-apoptotic [41]. Guan et al. [42] reported that conserved ELF2 isoforms, ELF2A and ELF2B, arising from alternative promoter usage, exerted opposing affects on target gene expression. Interestingly, ELF2A activated cancer cell proliferation, while ELF2B induced cancer cell apoptosis. Yang et al. also showed that receptor-interacting protein 3 (Rip3) was an apoptosis-inducing member, but the Rip3γ isoform down-regulated Rip3-mediated apoptosis in colon and lung cancers [43]. These findings suggest that modulating splicing might redirect the fate of cancer cells.

## Clinical application of alternative splicing in cancer

### Drug resistance

**Fig. 3:**
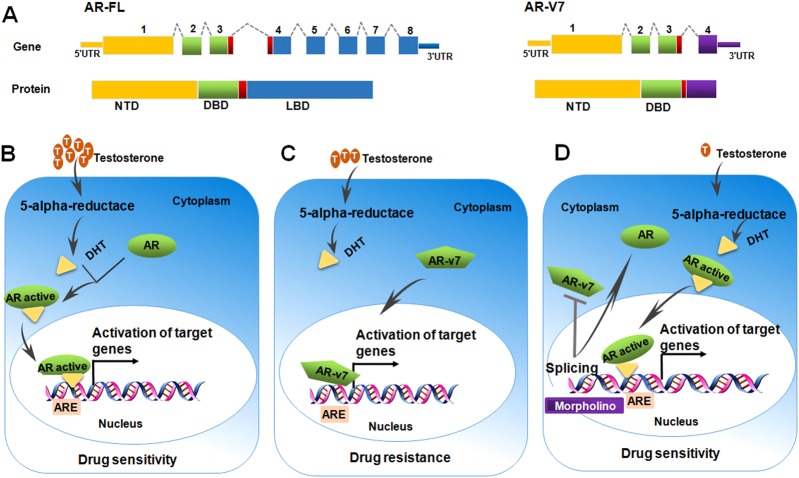
Schematic representation of AR-v7 mechanism of action in chemoresistance. **a** AR-FL is composed of an N-terminal domain (NTD), a central DNA-binding domain (DBD), a hinge region (red box), and a C-terminal ligand-binding domain (LBD). AR-v7 transcripts lack the reading frame for the LBD. **b** it highlights the AR activation axis, with conversion of testosterone to dihydrotestosterone (DHT) by the 5α-reductase enzyme, and subsequent AR activation, dimerisation, nuclear translocation and activation of transcriptional activation of target genes expression. **c** AR-v7 is characterised by loss of the LBD, often resulting in testosterone-independent activation of target genes expression. **d** morpholino technology caused a splice switch that inhibited expression of AR-v7 and blocked testosterone -independent growth of CRPC cells

RNA splicing might be an important determinant of clinical response and offered a therapeutic target for enhancing drug sensitivity in cancer (Table 1). One interesting gene that determines drug resistance is androgen receptor (AR) (Fig. 3). The full-length AR (AR-FL) is composed of an N-terminal domain, a central DNA-binding domain, a hinge region, and a C-terminal ligand-binding domain (LBD) (Fig. 3a). Because of insertions of cryptic exons downstream of the DNA-binding domain, AR-v7 transcripts lack the reading frame for the LBD. The LBD is the part of the AR molecule to which testosterone bind, as well as being the target of all antiandrogens including bicalutamide and enzalutamide [44]. This Fig. 3b highlights the AR activation axis, with conversion of testosterone to dihydrotestosterone (DHT) by the 5α-reductase enzyme, and subsequent AR activation, dimerisation, nuclear translocation and activation of transcriptional activation of target genes. AR-v7 is characterised by loss of the LBD with preservation of the DNA-binding domain, often resulting in testosterone-independent activation of the AR axis, a process known as constitutive activation (Fig. 3c). Multiple clinical correlative studies have suggested that AR-v7 is involved in the pathogenesis of disease progression, and poor outcomes from the use of abiraterone and enzalutamide in castration-resistant prostate cancer (CRPC) patients [45, 46]. AR-v7 functions as constitutively active transcription factor, thereby promoting resistance to AR-targeted therapies. Just then Seitz et al. also showed that testing of Ar-v7 mRNA levels in whole blood was a simple and promising approach to predict poor treatment outcome in CRPC patients receiving abiraterone or enzalutamide. AR-v7 has the potential to revolutionise how you make treatment decisions for patients with metastatic CRPC. Recently, therapies preventing expression of AR-v7 in CRPC were developed [47]. The splicing of AR isoforms AR-v7 is regulated by a single polyadenylation signal in AR intron 3. Blocking this signal with morpholino technology or silencing of the polyadenylation factor CPSF1 caused a splice switch that inhibited expression of AR isoforms and blocked androgen-independent growth of CRPC cells (Fig. 3c).

There were also another isoforms exhibited similar properties like AR-v7. Survivin-3b, an alternative splice isoform of survivin, increased the resistance of neoplastic cells to various chemotherapeutics [48]. Human BRCA1 played an important role in the repair of DNA-damage response, while BRCA1-Δ11 harbouring mutations in exon 11 of BRCA1 promoted chemoresistance to cisplatin [49]. Additionally, Haag et al. reported that both the long and the short isoform of the anti-apoptotic protein c-FLIP were critical regulators of apoptosis in pancreatic carcinoma cells and suppressed by chemotherapeutics [50]. A pre-treatment with drugs down-regulated c-FLIP isoform and rendered cells sensitive to chemotherapeutics [51].

### Radiosensitivity

The success of treating cancer patients by radiotherapy largely depends on tumour radiosensitivity. Several molecular factors that determine the sensitivity of tumour cells to ionising radiation showed alternative transcription initiation as a response to irradiation and have been identified during the last couple of years (Table 1). Through RNA-sequencing of normal and chemoradiation-resistant colon cancer cells, it was found that exon skipping was significantly increased in chemoradiation-resistant colon cancer cells [52]. Additionally, sCLU played a key role in pro-survival; the nCLU isoform took part in apoptosis in cancer treated with leptomycin B and irradiation [53]. A recent study demonstrated that the protein isoform Mcl-1L had an important role in survival and radioresistance of oral cancer patients [54]. An NPM2 isoform was able to increase cell survival after irradiation in cervical cancer cells [55].

**Fig. 4:**
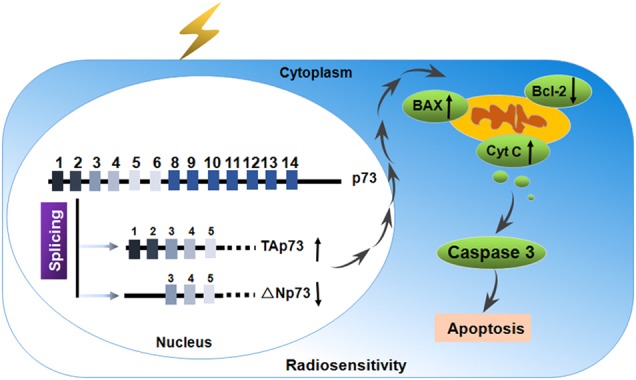
Schematic representation of p73 mechanism of action in radiosensitivity. The ratio of TAp73/ΔNp73 could be considered a potential molecular switch, regulating Bax/Bcl-2 ratio, and preventing cytochrome *c* release and caspase activation, and enabling sensitisation of cancer cells to radiation

One interesting molecular factor that determines the sensitivity of tumour cells to ionising radiation is p73 (Fig. 4). The p73 transcription factors are present in two forms, the full length Tap73 and the N-terminally truncated ∆Np73. High expression levels of ΔNp73 have been shown to strongly correlate with poor survival of cancer patients and ΔNp73 positive tumours show a reduced response to irradiation [56]. Liu et al. [57] suggested that p73a is an important determinant of cellular radiosensitivity in the p53-impaired cervical cancer cells, whereas up-regulation of ΔNp73 in cervical cancers was detected mainly in radioresistant cases. Our findings have been reported that there is a differential ΔNp73 expression in response to different LET radiations, and down-regulated ΔNp73 expression might play a critical role in sensitivity of tumour cells [58]. Finally, similar findings have been reported the anti-apoptotic ΔNp73 decreased in colon cancer cell lines (KM12C) exposed to γ-irradiation [59]. Our further research demonstrated that the ratio of TAp73/ΔNp73 could be considered a potential molecular switch, regulating Bax/Bcl-2 ratio, and preventing cytochrome c release and caspase activation, and enabling sensitisation of cancer cells to high linear energy transfer carbon beams [60, 61] (Fig. 4). Thus these findings suggested that p73 splicing was related to the radiosensitivity of cancer cells and may play an important role in the regulation of cellular radiosensitivity.

### Other therapies

Also for other potential therapeutic options, splice variations have been found to be relevant. Isoform Claudins18.2 (CLDN18.2) was targeted by the therapeutic antibody IMAB362, which was in advanced clinical phase II testing in gastric cancer patients [62]. Also, macrophage PI 3-kinase γ (PI3Kγ) controlled a critical switch between immune stimulation and suppression during inflammation and cancer [63]. Targeting PI3Kγ with a selective inhibitor, which is currently evaluated in a clinical trial (NCT02637531), could overcome resistance to immune checkpoint blockade in patients and promote cytotoxic tumour regression without targeting cancer cells directly [64]. Also the safety and pharmacokinetics of the PI3Kγ inhibitor IPI­549 is being explored, alone and in combination with an antibody against programmed cell death 1 protein [65].

## Options modulating RNA splicing in cancer

### SSOs as drugs for regulating RNA splicing in cancer

**Fig. 5:**
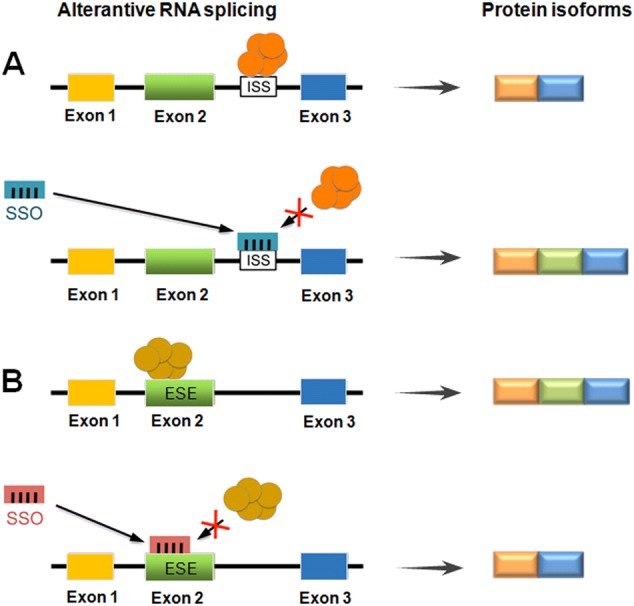
Splicing-switch oligonucleotides (SSOs) as a means to modulate RNA splicing. **a** An SSO that binds to an intronic splicing silencer (ISS) prevents binding of a negative splicing factor (orange), leading to exon inclusion. **b** An SSO that binds to an exonic splicing enhancer (ESE) blocks the binding of the stimulatory splicing factor (mustard), resulting in exon skipping

The use of RNA splicing modulators is an attractive option for establishing novel therapeutic cancer drugs [29]. To date, splicing-switch oligonucleotides (SSOs) were used as a means of drugs to modulate RNA splicing in cancer. SSOs are short, synthetic, antisense, modified nucleic acids and designed to base-pair and create a steric block to the binding of splicing factors to the pre-mRNA [66]. In this way, SSO base-pairing to a target RNA alters the recognition of splice sites by the spliceosome, which leads to an alteration of normal splicing of the targeted transcript. As shown in Fig. 5, SSOs can target and block trans-acting splicing factors that would change exon splicing and generate a new alternative protein isoform. Importantly, nucleotides of an SSO are chemically modified so that the RNA-cleaving enzyme RNase H is not recruited to degrade the pre-mRNA-SSO complex [67]. The modifications to the SSO have also been crucial to stabilise the SSO in vivo and improve cellular uptake and release as well as binding affinity [68]. SSOs were first described for the correction of aberrant splicing in human β-globin pre-mRNAs [69]. During the past 10 years, antisense oligonucleotide-mediated exon skipping and splice modulation have proven to be powerful tools for correction of mRNA splicing in genetic diseases. In 2016, the US Food and Drug Administration-approved Exondys 51 (eteplirsen) and Spinraza (nusinersen), the first exon skipping and exon inclusion drugs, to treat patients with Duchenne muscular dystrophy (DMD) and spinal muscular atrophy (SMA), respectively [70, 71]. The success of trials has also inspired therapeutic applications of SSOs in cancer (Table 2). The *Bcl-x* gene is alternatively spliced, expressing anti-apoptotic Bcl-xL and pro-apoptotic Bcl-xS. Bcl-xL expression is up-regulated in many cancers and considered a general mechanism by which cancer cells evade apoptosis. Mercatante et al. reported that Bcl-x SSOs shifted splicing from Bcl-xL to Bcl-xS in prostate and breast cancer cells in vitro. They also found that Bcl-xS protein induced by the SSOs sensitised the cancer cells to treatment with radiation and chemotherapeutic drugs [72]. In other studies, the same group demonstrated that Bcl-x SSOs delivered by lipid nanoparticles efficiently redirected Bcl-x pre-mRNA splicing and significantly reduced the tumour burden in mice with rapidly growing and highly tumorigenic lung metastases [73]. Soon after, Bauman et al. [74] presented the first demonstration of Bcl-x SSO efficacy in tumours in vivo. Recently, a Bcl-x SSO, modified using 2′-O-methoxyethyl-phosphorothioate and delivered with a cationic lipid into glioma and astrocyte cell lines, modulated the RNA splicing of Bcl-x pre-mRNA in glioma cell lines [75]. Table 2 provides examples of SSOs shown to be effective in the correction of cancer-related splicing isoform expression. This may represent a potential strategy for treating cancer. The potential of therapeutic SSOs is rather promising, with numerous clinical trials currently ongoing.

**Table 2:** SSOs as drugs for modulating RNA splicing in cancer

Target	Splicing events	Short description	Refs
ATM	Intron 28 inclusion	Susceptibility to radiation in HEK293 cell lines	[102]
Bcl-x	Exon 2 skipping	Induction of apoptosis in human glioma cell lines	[75]
BRCA1	Exon 11 skipping	Enhancing the effect of PARP inhibitors in breast cancer	[103]
HER2	Exon 15 skipping	Induction of apoptosis in breast cancer cells	[104]
HER4	Exon 26 skipping	Decrease growth of breast cancer cells	[105]
MDM2	Exon 11 skipping	Susceptibility to DNA damage in breast cancer cells	[106]
MDM4	Exon 6 skipping	Inhibite melanoma growth	[107]
PKM	Exon 10 inclusion	Susceptibility to chemotherapy in pancreatic cancer cells	[108]
STAT3	Alternative 3′ SS	Induction of apoptosis in breast cancer cells	[109]
WT1	Exon 5 inclusion	Kill HL60 leukaemia cells	[110]

## Small molecules as drugs for regulating RNA splicing in cancer

Additionally, small molecules as drugs have been developed that target RNA splicing in an effort to block the abnormal recruitment of splicing factors to mutant sequences [76]. Several natural compounds and synthetic derivatives as inhibitors have been reported to inhibit RNA splicing (Table 3). These inhibitors are cytotoxic at higher concentrations, although at lower concentrations these inhibitors were used as drugs for modulating RNA splicing in cancer. The earliest splicing modulators were FR901464 and its acetylated derivate spliceostatin A. In solid tumours, treatment with FR901464 reduced tumour size by 80% [29]. GEX1A and sudemycin D6 achieved a reduction of tumour size by 80 and 50%, respectively [77]. Similarly, pladienolide B was effective in reducing xenograft tumours by 60% [78]. The pladienolide B synthetic E7107 was tested in a phase 1 clinical trial with patients presenting different types of solid tumours [29]. However, due to the structural complexity of these molecules, their research and development has been significantly constrained. Recently, additional small molecule splicing inhibitors were identified, such as caffeine, digoxin, amiloride, sudemycins et al. In cervical cancer cells, caffeine and digoxin could reduce expression of the serine/arginine-rich splicing factor 3 (SRSF3) and increase expression of a p53b isoform. This change was accompanied by increased DNA damage and apoptosis [79]. Actually, caffeine and digoxin have been used in clinical application. Tang et al. suggested that 0.2 mM amiloride modulated cell radiosensitivity and RNA splicing of the apoptotic peptidase activating factor 1 (APAF1) [80]. 1.0 μm NB-506 mediated splicing inhibition by preventing complete phosphorylation of the SF2/ASF SR protein, affected apoptosis and tumour progression [81]. Madrasin at lower concentrations induced cell cycle arrest and apoptosis, and modulates splicing of multiple pre-mRNAs in cancer cells [82]. Sudemycins can regulate the production of alternatively spliced RNA transcripts and these alterations are more prevalent in tumour, as compared to normal cells, following drug exposure [83]. RNA-binding protein 39 (RBM39) is associated with pre-mRNA splicing factors; its inactivation by indisulam caused aberrant pre-mRNA splicing in many cancer cell lines [84]. Ibrutinib’s role in therapy was further expanded recently when the US Food and Drug Administration approved its use in both frontline and salvage treatment of patients with chronic lymphocytic leukaemia [85]. Recent clinical trials using selective pharmacologic targeting of CLDN18.2 and PI3Kγ have demonstrated their potential to control cancer [86]. These findings suggested that small molecules that act as a general inhibitor of splicing may represent a novel avenue for development of new anti-cancer agents.

**Table 3:** Small molecules as drugs for inhibiting RNA splicing in cancer

Target	Drugs	Short description	Refs
APAF1	Amioride	Radiosensitivity in glioblastoma multiforme cells	[80]
AR	6BIO	Drug sensitivity in prostate cancer cells	[111]
BTK	Ibrutinib	Target kinases in chronic lymphocytic leukaemia	[85]
RBM39	Indisulam	RBM39 degradation in the hematopoietic and lymphoid tissues	[84]
SF3b	Spliceostatin A	Display anti-proliferative effects in HeLa cells	[112]
SF3b	FR901464	Anti-tumour activity in lung cancer, breast cancer and et al	
SF3b	E7107	Block spliceosome assembly in patients with solid tumours	[113]
SF3b	Meayamycin B	Induction of apoptosis in head and neck cancer cells	[114]
SF3b	Jerantinine A	Anti-tumour activity by targeting splicing factor in breast cancer	[115]
SF3b	Sudemycin	Induce antitumour response in chronic lymphocytic leukaemia	[116]
SF3b	Pladienolides	Display anti-proliferative effects in HeLa cells	[117]
SF3b	GEX1A	Anti-tumour activity by targeting SAP155 protein in HeLa cells	[118]
Step 1	Isoginkgetin	Anti-tumour effect by splicing inhibition in HEK293 cell lines	[119]
Step 1	Madrasin	Modulate pre-mRNAs splicing in both HeLa and HEK293 cells	[82]
Step 1	Caffeine	Inhibit the survival of different types of tumour cells	[79]
SRSF3	Digoxin	Induction of apoptosis in HeLa cells	[120]
SR	NB-506	Phosphorylate SF2/ASF, affect apoptosis in leukaemia cell line	[81]

## Methodologies for detecting aberrant splicing isoform in cancer

The investigation of splicing changes in cancer has been shaped by the development of techniques (Table 4). For the relative quantification of isoform expression, real-time qPCR has been the gold standard for over a decade. More recently, droplet digital PCR is becoming widely implemented [87]. Nevertheless, identification of informative splice isoforms by this process is still laborious and the multiplexing capacity is limited. DNA-microarrays have provided a more high-throughput approach for studying transcriptomes. Zhang et al. [88] developed an assay based on junction microarrays and profiled alternatively spliced mRNA isoforms for prostate cancer classification. Another similar study on breast cancer by Lapuk et al. reported 181 candidate RNA splicing events in 156 genes [89]. Whole-genome exon arrays were used to study genome-wide RNA splicing events in colon, urinary bladder, prostate, breast and non-small cell lung cancer [90]. Furthermore, genomic tiling arrays provided an opportunity to systematically assess intron retention events arising from RNA splicing, which might be important for cancer [91]. A limitation of DNA microarrays is the fact that unknown transcripts or abnormal splicing isoforms cannot be detected. Recently, RNA-sequencing has become the state-of-the-art technology to determine splicing changes [92]. This is expensive but can be highly sensitive. Importantly, it can discover new isoforms without prior knowledge of the exact sequence. Nellore et al. produced the most comprehensive view of human transcriptome splicing to date [93]. For particular diseases, survey studies were performed, such as an analysis of pancreatic ductal adenocarcinoma, identifying 1354 genes that were expressed in RNA splicing isoforms [94]. Extensive studies have emerged on using RNA-Seq to study AS in cancer, and some studies are shown in Table 4. However, how to handle the massive data generated from RNA-Seq is still a major challenge to its applications.

**Table 4:** Overview of diagnostic methodologies of aberrant splicing isoforms in cancer

Level	Method	Material	Property	Refs
Transcript level	RT-PCR	Cell lines & tissues	Gold-standard for known gene	[61]
	Real time PCR			
	Digital droplet PCR		Single-cell capacity	[87]
	Exon junction array	Cancer cell lines	Genome-wide survey of splicing	[88]
	Exon array	Colon cancer; HNSCC	The GeneChip Human Exon array	[121]
	Tiling array	Breast cancer	Detection of intron retention	[91]
	RNA-seq	Different cancer types	Novel transcripts and gene fusions	[122]
	Primer Sequencing	Human heart & testes	Software of RT-PCR primers	[123]
Protein level	Quantitative imaging	Breast cancer	Combination of probes and imaging	[124]
	Peptidomics approach		Based on the PEPPI	[96]
	Western blotting	Different cancer types	Isoform-specific antibodies	[99]
	Immunohistochemistry			
	Immunofluorescence			

Comprehensive studies have also been performed at the protein level. For mass spectrometry, databases exist, in which the proteome-wide peptide content of isoform proteins is accessible that was computationally produced from sequence information so as to allow the detection of RNA splicing events at the proteome level [95]. PEPtidomics Protein Isoform Database, a comprehensive database of computationally synthesised human peptides was developed to study genetic variations and AS events at the proteome level. Zhang et al. [96] performed MS with PEPPI to analyse, identify and characterise novel AS isoform biomarkers from breast cancer. Another process for screening protein isoforms would be microarrays consisting of isoform-specific antibodies or other binder formats [97]. A limitation for this but also other analysis procedures is still the access to a set of binders that are sufficiently specific for the various protein isoforms. Nanobodies or entirely artificial structures such as DARPins [98] may be better suited to bind three-dimensional epitopes with high accuracy and thus discriminate better between protein isoforms. In another microarray-based approach, a process was developed by which the splice-isoform proteins are made accessible that are present in samples of individual patients without needing any prior knowledge [99]. This could represent a route to personal diagnosis, which eventually would translate to other platforms, however, which are better suited for routine use than protein arrays. Up to now, we still cannot do further research of some aberrant splicing isoforms. Firstly, we cannot find the commercial antibody. One reason is that the sequence similarity of isoforms is quite high. The other reason is that the different sequence between different isoforms is not fit to be antigen to make antibody. If we do not have the antibody, it is difficult to study the biological function in the protein level in the cancer cell. Although there is still a bottleneck in the research of alternative splicing in cancer, a more global picture of the role of alternative RNA splicing in human cancer is beginning to emerge.

## Concluding remarks

From a large body of experimental data, it has become increasingly clear that aberrant splicing isoforms play a critical role in cancer. While clinical trials utilising aberrant splicing isoforms profiling for patients prognosis and clinical response are now underway. Now we are at a point, at which a more comprehensive picture of the number and effects of alternative splicing as well as their part in the overall regulatory network begins to emerge, much work still needs to be done to provide sufficient information for translating this knowledge into clinical application. There are still many outstanding questions. Firstly, how specific are particular aberrant splicing isoforms and the resulting protein isoforms for cancer tissues? Secondly, what are the mechanisms by which small molecules modulate target gene splicing and thus inhibit tumour growth of human cancer? Thirdly, can protein isoforms that results from alternative RNA splicing in individual patients with cancer provide treatment options as part of a precision medicine approach? Last but not least, could splice variations be better reversed by applying new genome editing processes, such as CRISPR-Cas, or would proteomic procedures, such as provision of specific antibodies against the tumour-specific protein isoform do a better and/or technically superior job in clinical application? Overall, it becomes increasingly clear, that aberrant splicing isoforms plays a crucial role in cancer therapy. Although this would only be a beginning, it would represent a start into a new area of cancer therapy.
